# Automated, quantitative, low-pressure, cation-exchange chromatography of haemoglobin variants on midget columns

**DOI:** 10.1155/S1463924687000208

**Published:** 1987

**Authors:** R. S. Ersser, R. G. Drew, G. B. Barlow, M. Hjelm

**Affiliations:** Hospital for Sick Children and Institute of Child Health 30 Guilford Street London WC1N 1EH UK


Journal of Automatic Chemistry/*Journal of Clinical Laboratory Automation*, Vol. 9, No. 2 (April–June 1987), pp. 92–96

### Automated, quantitative, low-pressure, cation-exchange chromatography of haemoglobin variants on midget columns

**R. S. Ersser, R. G. Drew, G. B. Barlow and M. Hjelm**
*Hospital for Sick Children and Institute of Child Health, 30 Guilford Street, London WC1N 1EH*

### Introduction

Cation-exchange chromatography was a valuable but slow method for resolving mixtures of human haemoglobins [1], until the introduction of rigid, high-performance column packing materials [2 and 3]. The potential of midget columns (5–10 mm long) of polyaspartic acid bonded to silica spheres has been illustrated for the rapid separation of haemoglobin mixtures using simple equipment and without high pressure [4]. Stepwise changes in buffer composition were performed manually, as was quantitation by measurement of chart recorder peak areas. Suitable sampling and buffer mixing modules have been added to the original equipment [4] and a computer-operated chromatographic control and data acquisition package has been interfaced with the instrument to provide a fully automated, quantitative procedure.

The analytical performance of this prototype system when applied to the analysis of haemoglobin mixtures is reported. Results for mixtures containing abnormal and glycated haemoglobins are presented. A method suitable for screening neonates for some important haemoglobinopathies, using either umbilical cord blood or samples collected onto filter paper [5], has been devised.

### Materials and methods

### *Samples*

Blood samples from patients with known haemoglobinopathies were generously donated by the Department of Haematology, Central Middlesex Hospital, London NW10 7NS. Samples of umbilical cord blood were provided by the Department of Haematology, St Bartholomew's Hospital, London EC1A 7BE. Samples of blood collected onto filter paper were provided by the North Thames Neonatal Screening Service, Hospital for Sick Children, London. AFSC Haemocontrol was purchased from Helena Laboratories, Beaumont, Texas, USA and glycosylated haemoglobin controls from Pierce Chemical Company, Rockford, Illinois, USA. A mixture of blood from a cord sample containing haemoglobins Bart's, A, F and S and samples from adult patients containing A/S and A/C was used as an in-house control. Haemolysates of saline washed, red cells were prepared as previously described [4].

Two discs of 3 mm diameter were punched from blood spotted onto filter paper and placed together in a conical polypropylene tube (0·7 ml capacity). Potassium cyanide (0·01 M, 100 µl) was added, the tube shaken and, after 10 min, buffer A (400 µl) was added. The tube was mixed gently on a rotary mixer for a further 50 min, or allowed to stand in the refrigerator overnight. Carbon tetrachloride (100 µl) was added and, after vigorous vortexing (15 s), the tube was centrifuged (3000 rpm, 10 min). The tube was placed in the sampler. The moving probe of the sampler was adjusted to ensure that only the upper layer was aspirated into the instrument.

### *Reagents*

Buffer A contained 40 mM bis-tris (bis(2-hydroxyethyl)imino-tris(hydroxymethyl)methane) and 4 mM potassium cyanide adjusted to pH 6·2 with concentrated hydrochloric acid. Buffer B was Buffer A to which sodium chloride was added to a concentration of 200 mM prior to pH adjustment. Sodium chloride

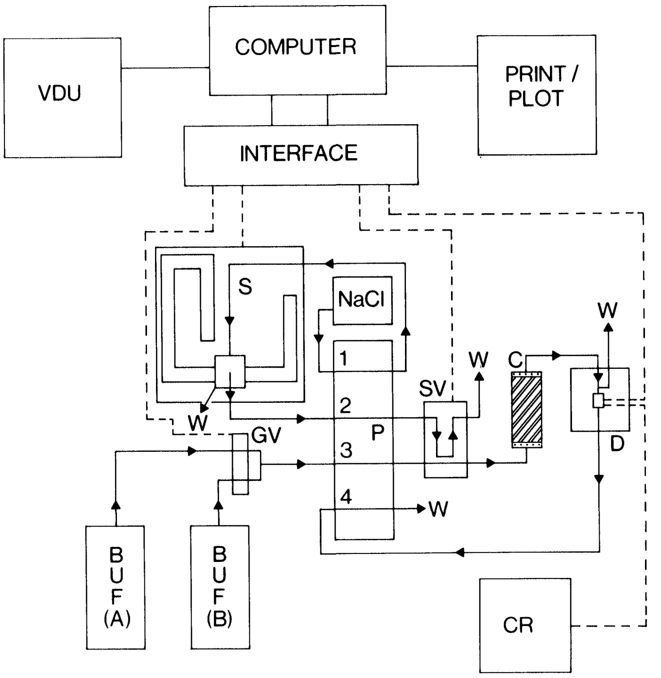

*Figure 1. Diagram of components of automated chromatographic system. Where C = column; CR = chart recorder; D = detector (420 nm); GV = gradient valve; S = sampler; SV = sample loading valves; W = waste; P = pump; 1 = 0·25 ml, 2 = 0·1 ml/min, 3 = 1·00 ml/min, 4 = 0·6 ml/min. --- electrical connections, – > – > – > liquid flow.*

(154 mM) was used in the sampler wash reservoir. This was replaced (manually) with deionized water for 20 min before the instrument was switched off, to prevent crystallization in the probe and fluid lines.

### *The instrument and its operation*

The component parts of the prototype instrument and their interconnection are illustrated in figure 1. The chromatographic modules used were: a four-channel, variable speed, peristaltic pump (Gibson Minipuls, Anachem Ltd, Luton, Bedfordshire, UK); a BEMAS autosampler, CF2 colorimeter and chart recorder (Burkard Scientific [Sales] Ltd, Uxbridge, Middlesex, UK); and miniature solenoid operated valves (Lee Products Ltd, Gerrards Cross, Buckinghamshire, UK) for sample loading and buffer proportioning. Operation was via an Apple IIe computer and a modified Apple pack 6 HPLC control and data system (Drew Scientific Ltd, Chiswick, London). The chromatographic modules were linked to the computer via a microprocessor-controlled interface (Drew Scientific).

The interface stored and commanded chromatographic instructions and performed data capture. During routine operation, the computer analysed the data and the VDU displayed the developing chromatogram, which was also presented graphically, along with the digital results, on the printer. The detector output was also monitored by the chart recorder.

Two floppy disk drives accommodated the system software and a third stored either final results or raw data for re-analysis with different mathematical parameters. The menu-driven computer program (Drew Scientific) included segments for: storage and editing of 10 gradient files, including auxillary instructions such as sample loading; similar facilities for data analysis methods and parameters; run sample and recalculation information; inspection of stored calibration data; and disk housekeeping.

The binary gradient profile could be divided into 25 segment changes in %B. Alterations in buffer composition were performed by controlling the fraction of time in each 6 s cycle, that buffer A was replaced by buffer B, using a subminiature solenoid operated valve (figure 1). Reproducible mixing of the alternative small segments of buffer occurred in the peristaltic pump tube (2·5 mm ID, trimmed to 26 cm long; purple/purple collar code) without an additional mixing chamber. The speed of the peristaltic pump was adjusted so that the column was

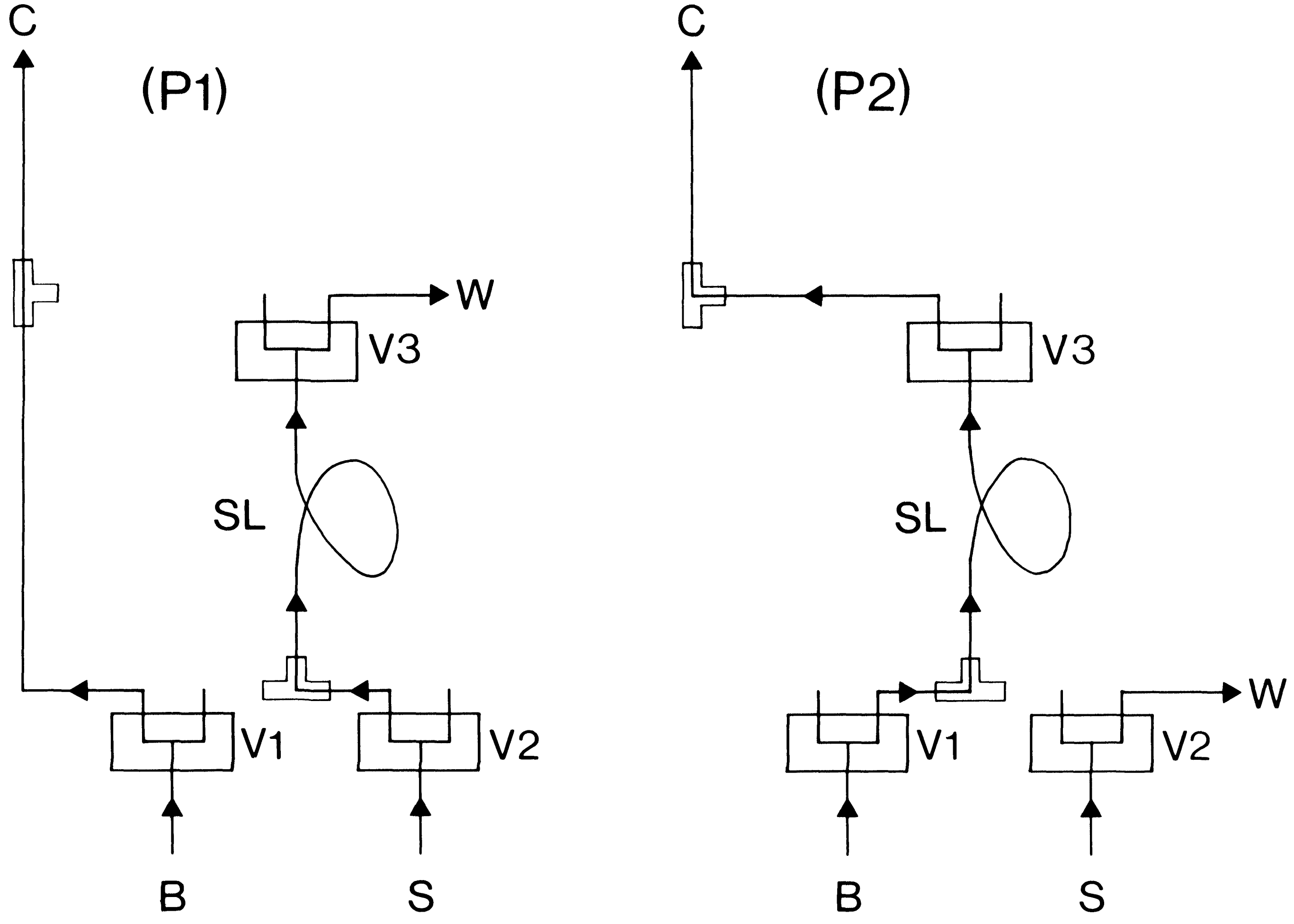

*Figure 2. Connections and operation of sample valves. Where B = buffer; C = to column; S = sample; SL = sample loop; V1, 2 and 3 = solenoid valves; W = waste. (P1)—sample loaded into loop, (P2)—sample loaded onto column.*

eluted at 1 ml/min. Tubes of suitable bores were selected to give the flow rates listed in figure 1 for the other solutions at this speed. Chromatography was performed on a 5 × 4·5 mm midget column [4] filled with poly-aspartic acid bonded to 10 μm spherical silica beads (Vydac) [6].

Up to 150 prepared haemolysates could be stored in the BEMAS autosampler. On each analytical cycle, following column equilibration, a sample, separated from the saline wash by both a leading and a following air bubble, was aspirated for 80 s (≡133 μl of diluted haemolysate) through the valve loader system (figure 2). Three subminiature solenoid operated valves were connected with minstac microbore tubing and fittings (Lee Products) as shown in figure 2, so that a calibrated loop (50 μl) could be filled with sample (P1). When the valves were switched to their opposite position (P2), column buffer was directed through the loop, and sample was swept onto the column. The valves were returned to their original configuration (P1) 20 s later. The BEMAS sampler probe remained in the saline wash for the rest of the analytical cycle, flushing the loop ready for the next sample.

The column outlet was connected to a flow-through colorimeter and the absorbance of the solution at 420 nm measured. A de-bubbling flow-cell of 10 mm path length was connected into the liquid flow circuit as illustrated in figure 1. Pulling only a portion (0·6 ml/min) of the eluate through the light path avoided stray gas bubbles passing the detector. The output of the colorimeter was connected to both the chart recorder, and the computer interface where the data was digitalized and either stored until convenient, or passed directly to the computer for analysis.

One of the 10 gradient files was used as a start-up program (10 min duration), loading a blank sample and using a steeply increasing gradient to check the mechanical performance of the system. A second file (20 min duration) was used as a shutdown program, after the last sample, to flush the sampler lines and column before switching off. In between, individual samples could be analysed by any of the eight remaining files. A calibration mixture was always run by the desired program before a batch of samples, to update elution times and check integration parameters. At the end of each analysis the % of each peak of the total area was printed. In this prototype instrument, peak identity was not automatic but was assigned by comparison with the relevant calibration run.

### Results

Typical chromatograms obtained using a program (27 min cycle) suitable for the routine detection of haemoglobinopathies are illustrated in figure 3. The coefficients of variation (CV) of peak elution times were between 0·135% (HbS) and 0·59% (HbF) within batch ($N = 11$); and between 0·334% (HbS) and 0·773% (HbF) between batches ($N = 21$) on three different columns. Within batch ($N = 11$) the CV of peak areas were 6·7% (Hb Barts), 2·54% (HbF), 2·66% (HbA), 2·84% (HbS) and 4·32% (HbC). Peak area versus amount of haemoglobin loaded was linear over the range 30–350 μg for each of four major haemoglobins ($N = 11$, HbF $r$ = 0·9977, HbA $r$ = 0·9947, HbS $r$ = 0·9974, HbC $r$ = 0·9952).

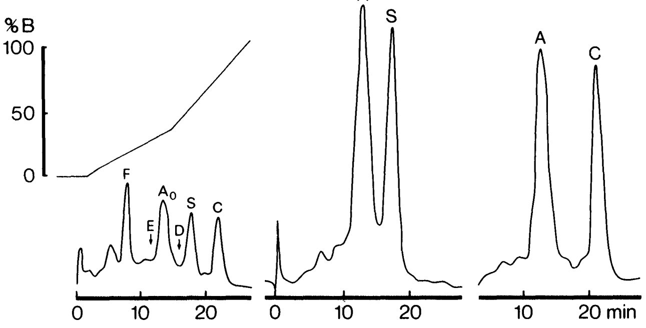

*Figure 3. Chromatograms of red cell haemolysates. Left to right: calibration mixture, heterozygote for sickle-cell disease, heterozygote for HbC.*

*Table 1. Results for reference haemoglobin mixtures analysed by midget column chromatography.*

| Haemoglobin | Midget column ($N$ = 10) % total x | dn-1 | cv % | Reference method ($N$ = not disclosed) % total x | Acceptable range * |
|---|---|---|---|---|---|
| HbF | 23·00 | 0·79 | 3·45 | 21·6 | 19–23 |
| $HbA_0$ | 34·43 | 0·45 | 1·27 | 39·1 | 34–45 |
| HbS | 22·40 | 0·53 | 2·36 | 20·9 | 20–22 |
| HbC | 19·56 | 0·31 | 1·56 | 18·4 | 15·5–21·5 |
| | | | | | ** |
| $HbA_{1c}$ | 5·99 | 0·32 | 5·34 | 5·6 | 4·9–6·5 |
| $HbA_0$ | 92·65 | 2·76 | 1·98 | 92·1 | 91·7–92·3 |
| $HbA_{1c}$ | 16·23 | 0·60 | 3·75 | 14·8 | 13·9–15·9 |
| $HbA_0$ | 79·89 | 2·95 | 3·67 | 81·4 | 81–82 |

* Helena AFSC haemocontrol analysed by scanning electrophoresis .
** Pierce glycated haemoglobin controls analysed by ion-exchange chromatographic methods.

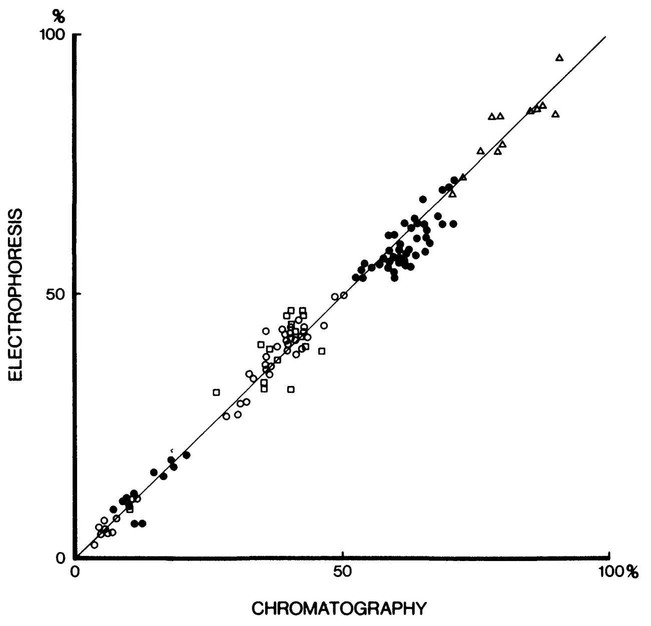

*Figure 4. Comparison of haemoglobin results obtained by chromatography and electrophoresis. Where △ = HbF, ● = $HbA_0$, ○ = HbS, □ = HbC.*

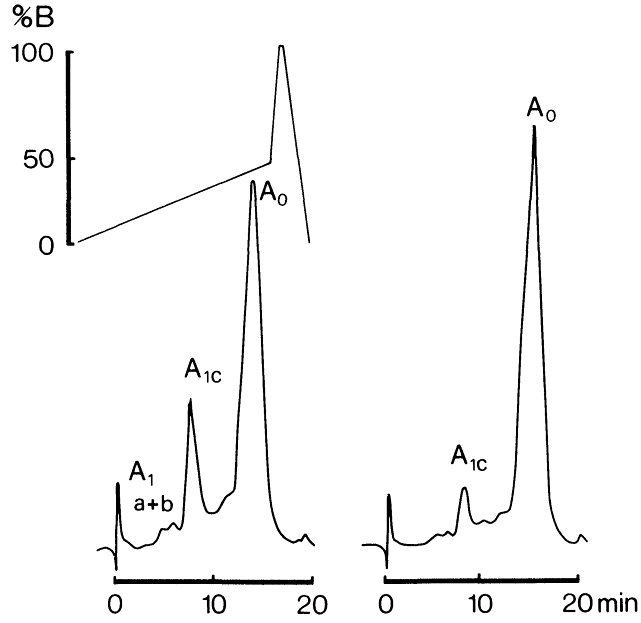

*Figure 5. Separation of glycated ($A_{1c}$) haemoglobin. Left: haemolysate from diabetic; right: haemolysate from normal subject.*

Results for a control sample independently assayed by scanning electrophoresis were within the manufacturer's acceptable limits (table 1). The lower value for HbA was related to a pre-HbF peak on the chromatogram (figure 3) which probably ran with HbA by electrophoresis.

A comparison of results obtained on haemolysates from patients with haemoglobinopathies, analysed by both the present method and scanning electrophoresis, is illustrated graphically in figure 4. Correlation coefficients ($r$) were; HbA $r = 0{\cdot}9813$ ($N = 58$), HbS $r = 0{\cdot}9876$ ($N = 40$), HbF $r = 0{\cdot}9117$ ($N = 12$), HbC $r = 0{\cdot}9281$ ($N = 18$). In both methods the contribution of minor constituents were ignored. The latter portion of the eluent profile could be accelerated when investigating diabetic patients, without haemoglobinopathies, for $HbA_{1c}$ levels (figure 5). Results obtained with calibration materials are presented in table 1. Chromatograms produced by a rapid (10·5 min cycle) program for the detection of the small amounts of several haemoglobins present in neonatal samples are illustrated in figure 6. Apart from a slight deterioration in reproducibility due to the gross concentration of HbF partially overlapping $HbA_0$, imprecision of peak times and areas was unchanged.

### Discussion

The performance of this prototype automated system was satisfactory and superior to the manual technique [4]. It compared favourably with the most commonly used routine method of electrophoretic separation on cellulose acetate, followed by spectrophotometric scanning of the bands (table 1, figure 4). Peak elution times within batch were adequate for identification, but were more variable between batch and between columns. This variation was minimized by running a calibration mixture before, during and after each batch of samples.

A virtue of the multifile gradient system is that programs for specific purposes can be stored and re-called when required. Additionally, samples can be analysed by different files within the same batch. The separation illustrated in figure 3 is equivalent to the most generally useful profile produced by manual stepwise changes in buffer [4]. Both major and minor components are resolved. Haemoglobins J, D and E were detected but a shallower gradient (40 min cycle) was required for positive identification and quantitation of these variants. Haemoglobin $A_{1c}$ was analysed with similar specificity and accuracy to the HPLC techniques (table 1), but at a slower rate of analysis. The fast program used for neonatal studies (figure 6) could be used for analysis of total $HbA_1$ content in older patients.

The quantitative analysis of small amounts of haemoglobin $A_2$ performed on long (50–200 mm) columns of polyaspartic acid [3] could not be repeated on midget columns. It could not be completely resolved from the major $HbA_0$ peak with buffers containing sufficient salt to produce a definite peak of the minor component. Therefore it could not be quantitated with the precision or accuracy necessary to detect heterzygotes for Thalassaemia.

An accelerated separation was devised specifically for the detection of sickle-cell disease in the neonatal population (figure 6). Either cord blood or samples collected onto filter paper for mass-screening programmes [5] could be analysed. Accurate measurement of small concentrations of $HbA_0$, HbS (and HbC) was possible in the presence of the gross amount of HbF characteristic of these specimens. Homozygotes (HbF/HbS) were distinguished from heterozygotes (HbF/$HbA_0$/HbS) as the presence or absence of $HbA_0$ was clearly apparent (figure 6). This separation is less certain by electrophoresis on cellulose acetate [7]. The correct assignment of over 100 cord

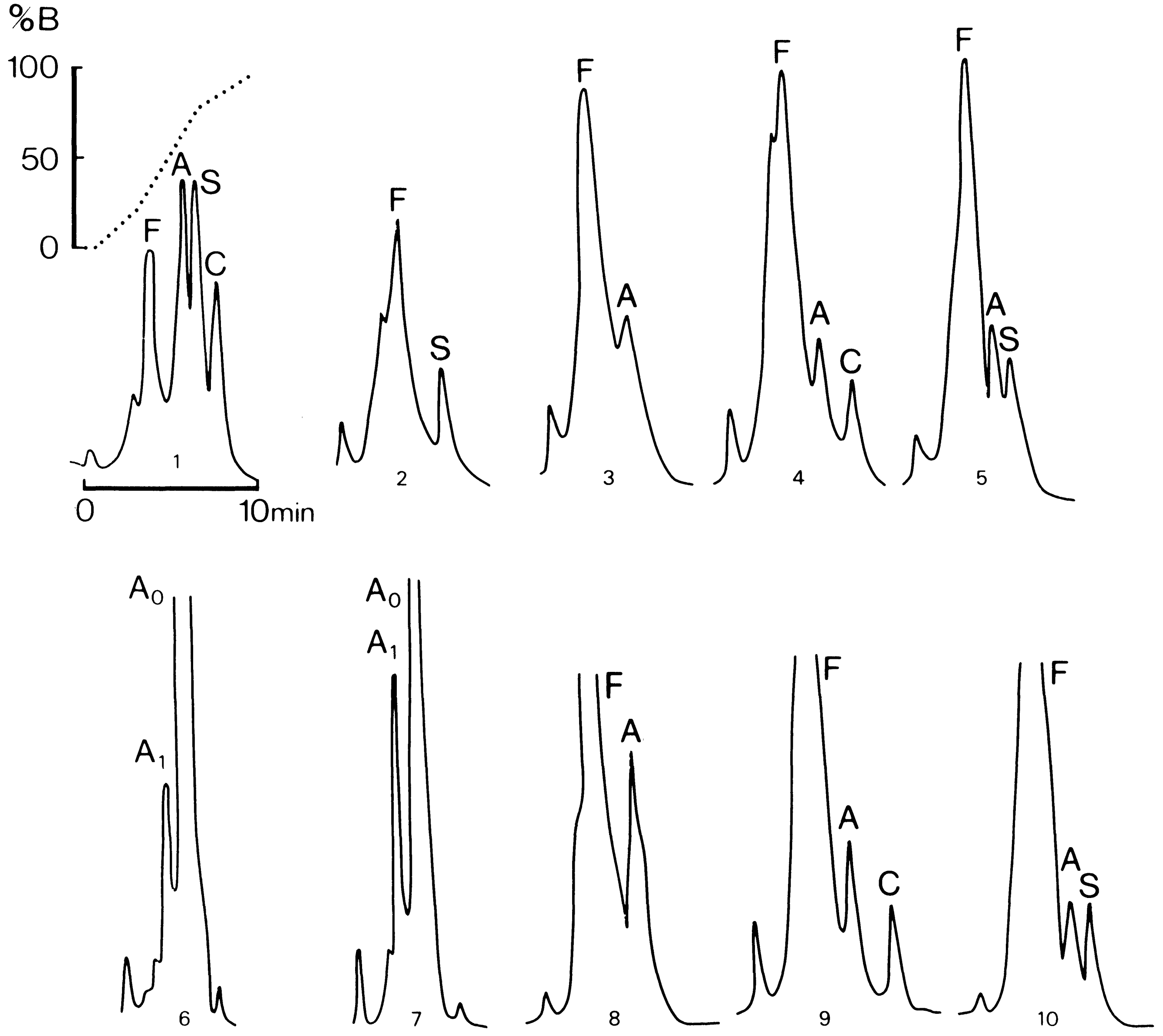

*Figure 6. Typical chromatograms obtained using a rapid elution system suitable for neonatal screening. Where 1 = calibration mixture; 2–5 = eluted neonatal blood spots from homozygote for HbS, homozygote normal, heterozygote for HbC and heterozygote for HbS; 6 = red cell haemolysate from normal adult, 7 = red cell haemolysate from adult diabetic, 8–10 = red cell haemolysates from cord bloods equivalent to subjects 3–5.*

bloods and 1000 blood spot samples has been achieved by this method. Detailed results of a survey at present under investigation will be published elsewhere.

### Acknowledgements

Ms J. Henthorn of the Central Middlesex Hospital, Ms B. Wilde of St Bartholomew's Hospital and Ms P. Lilley of the North Thames Neonatal Screening Unit provided the samples analysed in this study and offered much valuable advice. This work was in part funded (RGD) by the North East Thames Regional Research Fund.

### References

1. Dozy, A. M. and Huisman, T. H. J., *Journal of Chromatography*, **40** (1969), 62.

2. Ou, C. N., Buffone, G. J., Reiner, G. L. and Alpert, A. J., *Journal of Chromatography*, **266** (1983), 197.

3. Turpeinen, U., *Clinical Chemistry*, **32** (1986), 999.

4. Ersser, R. S., Barlow, G. B., Drew, R. G. and Hjelm, M., *Biomedical Chromatography* (in press).

5. Garrick, M. D., Dembure, P. and Guthrie, R., *New England Journal of Medicine*, **288** (1973), 1265.

6. Alpert, A. J., *Journal of Chromatography*, **266** (1983), 23.

7. Griffiths, K. D., Raine, D. N. and Mann, J. R., *British Medical Journal*, **284** (1982), 933.

